# An antibody Fc engineered for conditional antibody-dependent cellular cytotoxicity at the low tumor microenvironment pH

**DOI:** 10.1016/j.jbc.2022.101798

**Published:** 2022-03-03

**Authors:** Yutong Liu, Alison G. Lee, Annalee W. Nguyen, Jennifer A. Maynard

**Affiliations:** 1 Departments of Chemical Engineering, University of Texas, Austin, Texas, USA; 2 Departments of Molecular Biosciences, University of Texas, Austin, Texas, USA

**Keywords:** mammalian display, protein engineering, histidine scanning, antibody-dependent cellular cytotoxicity, pH selectivity, conditional activity, tumor microenvironment, antibody engineering, ADCC, antibody-dependent cellular cytotoxicity, ADCP, antibody-dependent cellular phagocytosis, BLI, biolayer interferometry, BSA, bovine serum albumin, CHO, Chinese hamster ovary, CI, confidence interval, EC_50_, 50% effective concentration, FACS, fluorescence-activated cell sorting, FBS, fetal bovine serum, FcRn, neonatal Fc receptor, K_d_, equilibrium dissociation constant, NK, natural killer, SA, streptavidin, VISTA, V-domain immunoglobulin suppressor of T cell activation

## Abstract

Despite the exquisite specificity and high affinity of antibody-based cancer therapies, treatment side effects can occur since the tumor-associated antigens targeted are also present on healthy cells. However, the low pH of the tumor microenvironment provides an opportunity to develop conditionally active antibodies with enhanced tumor specificity. Here, we engineered the human IgG1 Fc domain to enhance pH-selective binding to the receptor FcγRIIIa and subsequent antibody-dependent cellular cytotoxicity (ADCC). We displayed the Fc domain on the surface of mammalian cells and generated a site-directed library by altering Fc residues at the Fc–FcγRIIIa interface to support interactions with positively charged histidine residues. We then used a competitive staining and flow cytometric selection strategy to isolate Fc variants exhibiting reduced FcγRIIIa affinities at neutral pH, but physiological affinities at the tumor-typical pH 6.5. We demonstrate that antibodies composed of Fab arms binding the breast cell epithelial marker Her2 and the lead Fc variant, termed acid-Fc, exhibited an ∼2-fold pH-selectivity for FcγRIIIa binding based on the ratio of equilibrium dissociation constants K_d,7.4_/K_d,6.5_, due to a faster dissociation rate at pH 7.4. Finally, *in vitro* ADCC assays with human FcγRIIIa-positive natural killer and Her2-positive target cells demonstrated similar activities for anti-Her2 antibodies bearing the wild-type or acid-Fc at pH 6.5, but nearly 20-fold reduced ADCC for acid-Fc at pH 7.4, based on EC_50_ ratios. This work shows the promise of mammalian cell display for Fc engineering and the feasibility of pH-selective Fc activation to provide a second dimension of selective tumor cell targeting.

Antibody therapeutics have revolutionized cancer treatments by specific recognition of a tumor-associated antigen through the Fab-binding site, with protection often mediated by Fc recruitment of immune cells. However, since the tumor-associated molecules targeted can also be present on healthy tissues, many antibody therapeutics exhibit undesirable side effects due to immune activation at nondisease sites. These “on-target, off-tumor” effects have been reported for a number of monoclonal antibody therapies. For example, the anti-vascular endothelial growth factor bevacizumab disrupts tumor angiogenesis during treatment of lung, kidney, breast, brain, and colorectal cancers but also causes proteinuria in ≤63% of patients and hypertension in ≤36% of patients (1). For the anti-epidermal growth factor receptor cetuximab, approved for the treatment of colorectal and skin cancers, various skin disorders arise in a high percentage of patients (2). During treatment of Her2+ breast cancer with the anti-Her2 trastuzumab, clinical results have shown a clear correlation between treatment and impairment of the left ventricular ejection fraction (3, 4), resulting in cardiac dysfunction. These complications can lead to a reduced tolerance for and even discontinuation of therapy (5).

In addition to expressing tumor-associated antigens, tumors also alter their local tissue environments, which present opportunities for tumor targeting *via* characteristics orthogonal to antigen specificity. For example, matrix metalloproteases degrade extracellular matrix components to support tumor invasion into surrounding tissues. Accordingly, matrix metalloprotease inhibitors are progressing as antimetastatic agents in clinical trials (6). Similarly, solid cancers generate local microenvironments with dysregulated pH regardless of the tissue origin or genetic background (7, 8). This is a direct result of the high proliferative and glycolytic rates characteristic of cancer cells, which generate more lactate and protons than normal cells, known as the Warburg effect (9). To maintain a neutral intracellular pH, these cationic species are pumped out of the cells, resulting in a lower extracellular pH compared to nontumor tissues (7). The typical pH of tumor tissues ranges from 6.5 to 6.9, with values as low as 5.85 reported (10, 11), while that of normal-tissue cells is 7.2 to 7.5 (8, 12). Acidosis seems to occur very early in tumor formation (13), with recent reports observing low pH proton “halos” surrounding a single tumor cell (14), suggesting that even micrometastases will be characterized by locally low pH values.

The pH difference between normal and cancerous tissues offers a potential opportunity to improve antibody specificity for cancerous cells and reduce toxicities toward normal cells. Protein engineering of pH-dependent antigen binding has been reported for an anti-Her2 antibody (15), but paratope engineering is limited to an individual antibody targeting a single antigen. By contrast, antibody effector functions are highly dependent on interactions between the conserved Fc and immune receptors. Binding of the antibody Fc to FcγRIIIa on natural killer (NK) cells activates antibody-dependent cell-mediated cytotoxicity (ADCC), which is reported to be the major mechanism of action for several FDA-approved monoclonal antibodies (16). The Fc–FcγRIIIa binding affinity is known to impact clinical efficacy: individuals expressing the FcγRIIIa V158 allele with high Fc affinity (K_d_ ∼ 200–500 nM) exhibit superior responses to antibody therapeutics than those carrying the low affinity F158 allele (K_d_ ∼ 850–4500 nM) (17, 18). Moreover, clinical results with the recently approved margetuximab, an anti-Her2 antibody derived from the same 4D5 parent antibody as trastuzumab but bearing a modified Fc domain with stronger FcγRIIIa binding and improved ADCC, revealed more frequent adverse events for patients receiving margetuximab than trastuzumab (19). This suggests that Fc variants with higher FcγRIIIa affinity may exacerbate off-target effects unless immune activities are restricted to the tumor microenvironment.

To generate a broadly applicable pH-selective targeting strategy, we aimed to develop Fc variants with selective ADCC activity in the acidic tumor microenvironment. To evaluate the feasibility of this approach, we engineered the human IgG1 Fc domain to retain physiological FcγRIIIa affinity at the low tumor tissue pH but have weaker affinity at the neutral pH of normal tissue. We generated and screened an antibody Fc library in a mammalian cell display platform, which allowed for native glycosylation of the Fc and high-throughput Fc selection. Since antibody Fab and Fc domains can be combined in a modular fashion, the acid-Fc reported here could be combined with Fab arms binding any antigen that would benefit from pH-selective targeting.

## Results

### Chinese hamster ovary cell display discriminates among Fc variants with different FcγRIIIa affinities

**Figure 1:**
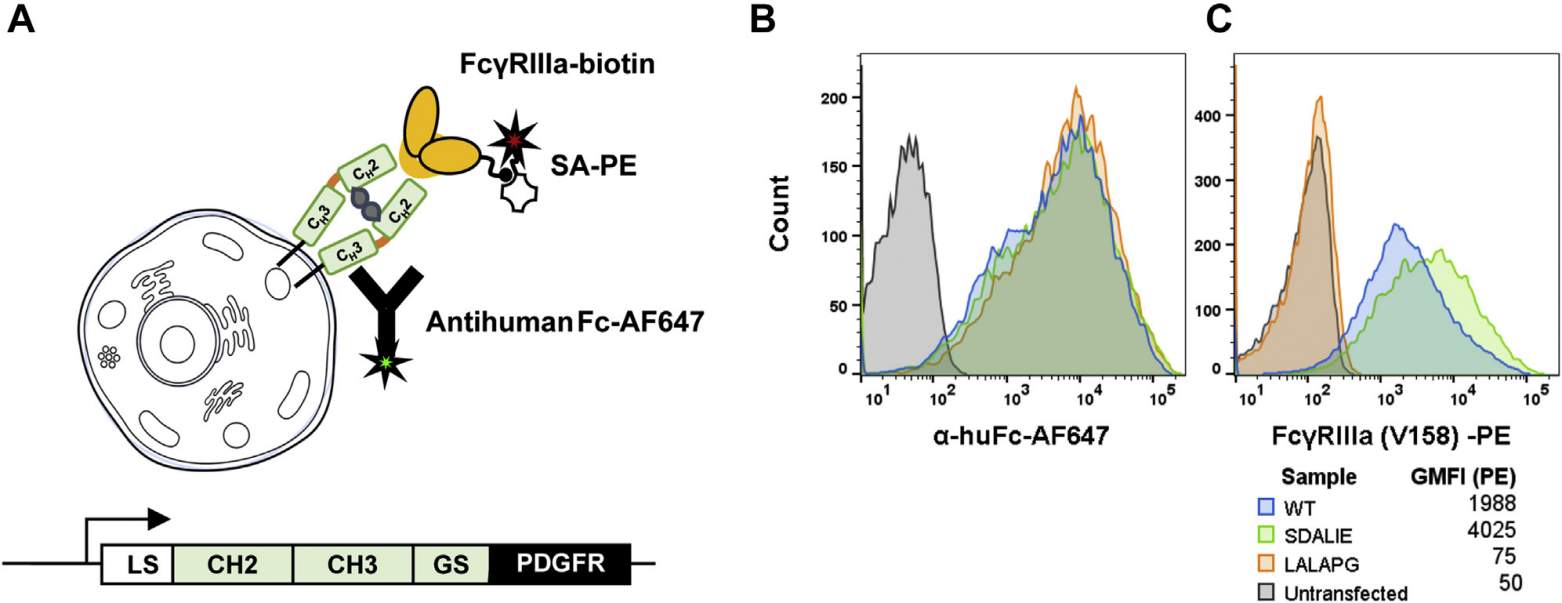
**Display of human IgG1 Fc on the CHO cell surface.***A*, schematic of the Fc CHO display construct and the staining strategy. The human IgG1 hinge CH2 and CH3 regions were appended with an N-terminal murine IgK secretory leader sequence (LS), C-terminal (Gly_3_Ser)_2_ linker (GS), and PDGFR transmembrane domain and introduced into the pPyEBV vector. The wild-type human IgG1 Fc (WT), an Fc variant with impaired binding to FcγRIIIa (LALAPG), and an Fc variant with enhanced binding to FcγRIIIa (SDALIE) were transfected into CHO cells, stained, and assayed for (B), Fc display level with anti-human Fc-Alexa Fluor 647 (AF647) and (C), binding to biotinylated FcγRIIIa (allele V158) monomer conjugated to streptavidin-PE *via* flow cytometry. Untransfected controls are also shown; the data are representative of three experimental repeats. CHO, chinese hamster ovary.

For Fc engineering, we selected a mammalian cell display system, which allows for engineering on the same cell line used for manufacturing (20, 21). Chinese hamster ovary (CHO) cells preserve the essential glycosylation at position N297 that supports binding to classical Fcγ receptors and can modulate Fc effector functions independently of amino acid residue changes (22). To first determine the display level and functionality of Fc proteins expressed on the CHO cell surface, we cloned residues 216 to 447 (EU numbering) of the human IgG1 Fc domain, corresponding to the complete hinge, CH2 and CH3 domains, with an N-terminal murine IgK leader sequence into the pPyEBV vector we previously used for Fab and TCR display on CHO cells (20, 21) (Fig. 1*A*). The expressed homodimeric Fc is anchored to the CHO cell surface by a (Gly_3_Ser)_2_ linker and PDGFR transmembrane region at the C-terminal end of the CH3 domain. As previously (20), we used a modified Kozak sequence to reduce Fc expression level (23) and modulate avidity effects.

The wild-type human IgG1 Fc and known variants with greatly reduced (LALAPG) (24) or improved (SDALIE) (25) FcγRIIIa binding were cloned into the display construct. After sequence confirmation, purified plasmid DNA was transiently transfected into CHO-T cells to allow plasmid maintenance for ∼8 weeks. After hygromycin-B selection, Fc display levels were monitored by antihuman Fc-Alexa Fluor 647 (AF647) and biotinylated FcγRIIIa allele V158 monomerically bound to streptavidin (SA)-PE (Fig. 1*A*). Staining with antihuman Fc and FcγRIIIa was performed separately to avoid interference between the receptor and the anti-Fc antibodies. Flow cytometry showed similar high display levels for all three Fc variants on the surface of CHO cells (Fig. 1*B*). Consistent with the reported affinities (24, 25), SDALIE showed higher FcγRIIIa staining than wild-type, while LALAPG showed no FcγRIIIa staining at all (Fig. 1*C*). These results indicate that our system displays functional Fc variants and distinguishes among Fcs with known FcγRIIIa affinity differences. Accordingly, this system should be suitable for selection of Fc variants with different FcγRIIIa-binding characteristics.

### Creation of an Fc library targeting the CH1-CH2 hinge region

Previous efforts to engineer pH-sensitive protein–protein interactions guided this work. The naturally pH-dependent interaction between human IgG1 Fc and the neonatal Fc receptor (FcRn) has been engineered to adjust antibody *in vivo* half-lives (26, 27, 28), while novel pH-dependent binding has been introduced into other binding partners *via* histidine scanning mutagenesis (15, 29). In both cases, pH sensitivity relies on the presence of ionizable histidines in the binding interface, whose pK_a_ of ∼6.0 can be modulated by adjacent residues. When histidines within the paratope and/or epitope are protonated by an acidic environment, they can mediate interactions with negatively charged or polar residues on a binding partner; these interactions are lost at neutral pH when histidines are not protonated.

**Figure 2:**
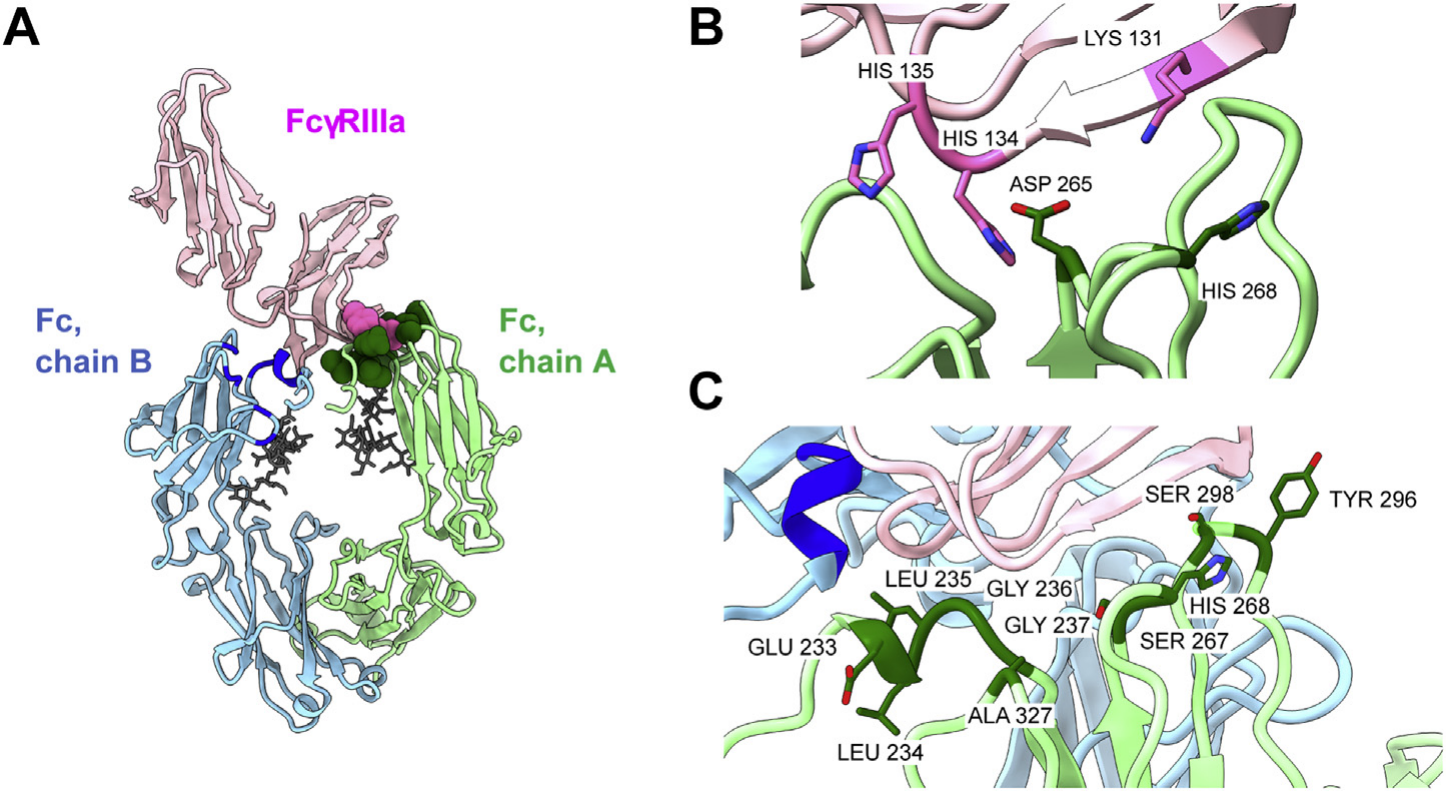
**Structural interface between Fc and FcγRIIIa.***A*, the crystal structure of human Fc complexed with FcγRIIIa is shown (PDB 3SGJ). The two Fc homodimer chains are in *green* (chain A) and *blue* (chain B), with FcγRIIIa in *pink* and the Fc glycosylation shown as *gray* sticks. FcγRIIIa residues H134 and H135 are shown as *hot pink spheres*. The Fc residues altered in the library (Table 1) are shown in *dark green spheres* on chain A only for clarity, with the backbone of the altered residues on chain B highlighted in *dark blue*. Residues L234, L235, G236, G237, S267, A327 are within <6.5 Å of FcγRIIIa H134 and H135 and were altered to acidic residues, while chain A residues E233, Y296, S298 are <5 Å of polar FcγRIIIa residues and were altered to histidine. The only Fc histidine in the interface, H268, was allowed to remain or be replaced with Y/A/D/S. *B*, the side chain of the existing histidine residues in the Fc-FcγRIIIa interface are shown along with Fc D265 that forms hydrogen bonds with H134. The only FcγRIIIa residue <5 Å of Fc H268, K131, is also shown. *C*, the Fc residues selected for the library design are highlighted on chain A. Molecular graphics and analyses performed with UCSF ChimeraX (66, 67).

The Fc–FcγRIIIa crystal structure (30, 31) shows an asymmetric FcγRIIIa footprint on the Fc homodimer near the CH1-CH2 hinge region (Fig. 2*A*). The Fc–FcγRIIIa interactions are dominated by van der Waals contacts, including P329 on one chain (here called chain B), which forms a “proline sandwich” with W90 and W113 of the receptor (30). However, ∼6 potential hydrogen bonds are also present (30), primarily involving the other Fc chain (here called chain A), which may be amenable to engineering for pH-selective binding. Notably, if FcγRIIIa approaches the opposite Fc face, these interactions are reversed with chain B dominating the charge interactions and chain A participating in the “proline sandwich”. The chain A-receptor interface includes two FcγRIIIa histidine residues (H134 and H135) and one Fc histidine (H268; Fig. 2*B*). Residues H134 and H135 are in close proximity to multiple Fc residues, with H134 able to hydrogen bond with D265. Fc residue H268 is near FcγRIIIa K131, but no electrostatic interactions form between these residues.

To support the formation of new charge–charge interactions, we selected six Fc residues (L234, L235, G236, G237, S267, and A327, Fig. 2*C*) within 6.5 Å of the FcγRIIIa histidines. These were allowed to remain unchanged or to be substituted with negatively charged glutamic or aspartic acid, residues with pK_a_ values near 4 that likely retain negative charges at tumor-typical pH values. To introduce new histidine residues, we identified three Fc residues within 5 Å of polar FcγRIIIa residues (E233, Y296, and S298, Fig. 2*C*) for histidine scanning, while the existing H268 (Fig. 2*C*) was allowed to remain a histidine or be substituted with Y/A/D/S to cover the chemical diversity compatible with protein–protein interactions with few codons (32).

This diversity was introduced into the Fc gene using primers with degenerate codons and overlap-extension PCR. At some sites, the degenerate codons introduced additional diversity beyond the intended changes (Table 1), resulting in a theoretical library size of 6 × 10^6^ (DNA) and 1.1 × 10^6^ (protein) variants. Amplified Fc genes were ligated into the pPyEBV vector containing an Fc with a premature stop codon to prevent expression from background plasmid and transformed into *Escherichia coli* to achieve an actual library size of ∼1 × 10^7^ transfectants. Sequencing of 10 colonies revealed 10 unique DNA sequences with three containing frameshifts, as is typical for PCR-generated libraries, and no unmodified background sequences, indicating that the actual library size is similar to the theoretical DNA library size. The designed primers allowed for simultaneous mutations, and the seven intact sequences each contained more than five different mutations.

**Table 1:** Antibody Fc domain amino acid sequences

Residue #	233	234	235	236	237	267	268	296	298	327
Wild-type	E	L	L	G	G	S	H	Y	S	A
Library	**H** **Q** **D** E	**E** **D** **Q** **H** **V** L	**E** **D** **Q** **H** **V** L	**E** **D** G	**E** **D** G	**E** **D** **K** **G** **N** **R** S	**D** **S** **Y** **A** **P** H	**H** Y	**H** **N** **R** S	**E** **D** A
3A	**D**	**V**	**V**	G	G	**E**	**D**	**H**	S	A
3E	**D**	**V**	**V**	G	G	**D**	**D**	Y	**R**	A
3F	**D**	**V**	**D**	G	G	**G**	**D**	**H**	S	A
4A	**D**	**V**	**D**	G	G	**E**	**D**	**H**	S	A
Acid-Fc (3A2/4A2)	E	L	L	G	G	**E**	**D**	**H**	S	A
3E2	E	L	L	G	G	**D**	**D**	Y	**R**	A
3F2	E	L	L	G	G	**G**	**D**	**H**	S	A

Shown are the sequences for wild-type (WT) human IgG1 Fc, the residues allowed at each targeted position during library design and the changes present in selected Fc variants. Wild-type residues are represented in regular font, while introduced changes are shown in bold.

### Identification of Fc variants with pH-selective binding

**Figure 3:**
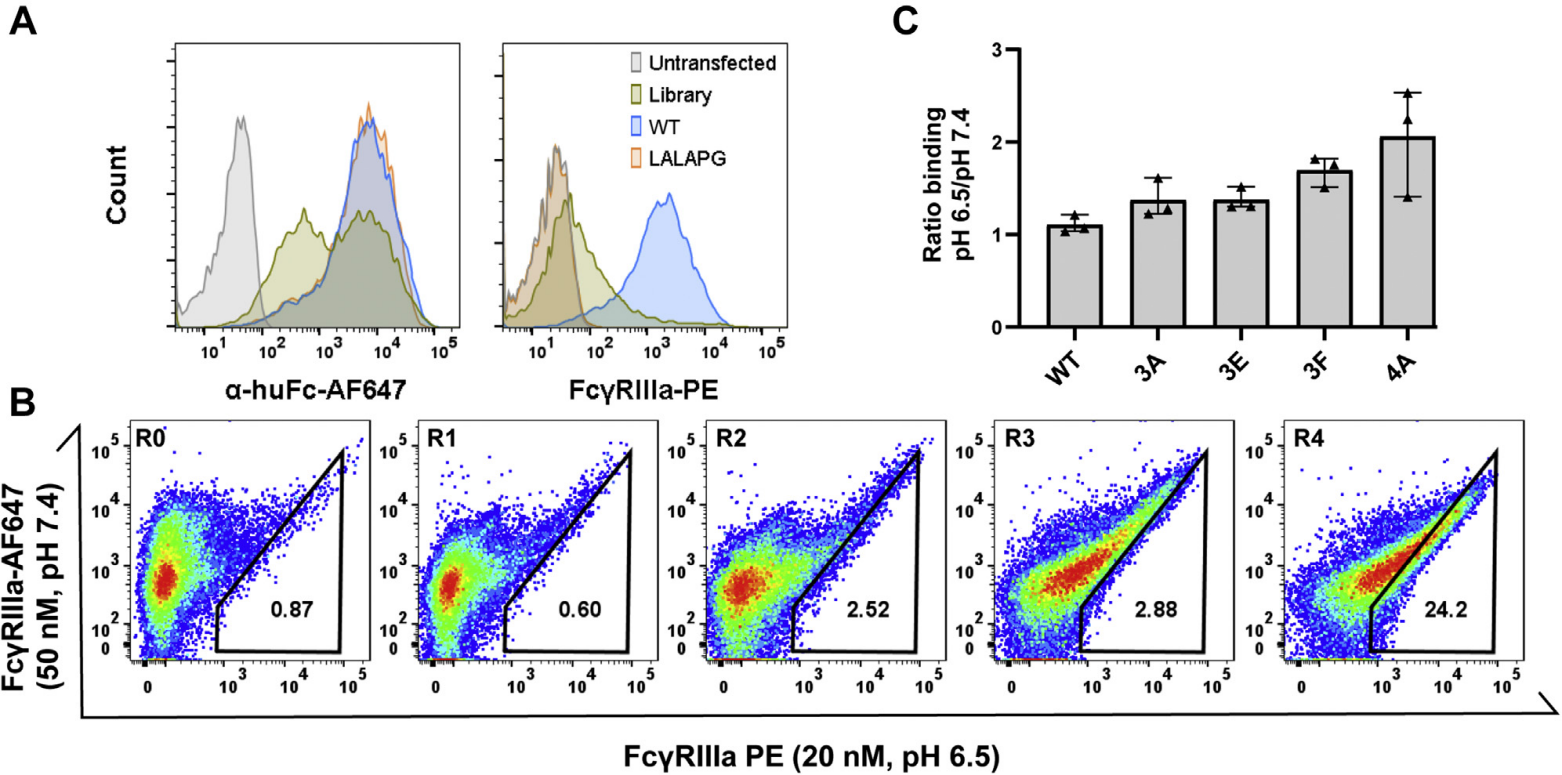
**Fc variants with pH-dependent binding to FcγRIIIa isolated from CHO display library.***A*, the Fc display library was transfected into CHO-T cells and Fc display level monitored by antihuman Fc-AF647 and FcγRIIIa binding monitored by monomeric FcγRIIIa-SA-PE. *B*, the library was sorted over two rounds for binding to FcγRIIIa-SA-PE at pH 6.5 (first round at 50 nM, second round at 20 nM), followed by two rounds of enrichment for stronger pH-dependence. To select for pH-dependent binding, the library was stained first with 50 nM FcgRIIIa-SA-AF647 at pH 7.4, washed, and then stained with 20 nM FcγRIIIa-SA-PE at pH 6.5. The gate shown is representative of the sorting gate used in round four. *C*, individual clones selected during rounds three and four were isolated and sequenced before transfection into fresh CHO-T cells, staining as above, and assessment of Fc variant display and FcγRIIIa binding at pH 6.5 and pH 7.4. The pH-selectivity of each variant was calculated as the percent of cells binding FcγRIIIa at pH 6.5 divided by percent of cells binding FcγRIIIa at pH 7.4. The data shown are pooled from three experimental replicates; these data collected with FcγRIIIa allele V158.

The library was transfected into 4.5 × 10^7^ CHO-T cells with carrier plasmid as previously described (20) to ensure each cell expressed at most one Fc variant and representation of every library member. Assuming a 30% transfection efficiency, which we typically observe for this system, ∼2 copies of each *E. coli* transfectant were present in the final CHO cell library. After hygromycin B selection, the cells were stained with antihuman Fc-AF647 and monomeric PE-labeled FcγRIIIa (V158) separately at neutral pH and scanned by flow cytometry. Many changes introduced into the Fc region are likely detrimental to Fc expression, folding, or FcγRIIIa binding. Consistent with this expectation, the library exhibited bi-phasic high and low Fc display levels. Most library variants lost binding to FcγRIIIa, although a long tail overlapping with the FcγRIIIa binding profile for the wild-type Fc suggested some members retain strong FcγRIIIa binding (Fig. 3*A*).

The library (7 × 10^6^ CHO cells) was sorted by fluorescence-activated cell sorting (FACS) for two rounds to first isolate clones retaining binding to the high affinity FcγRIIIa allele V158 at pH 6.5. The library was then subjected to a dual-color staining process for two additional sorting rounds to enrich for clones with stronger FcγRIIIa binding at pH 6.5 than at pH 7.4 (Fig. S1). In this process, the cells were first labeled with 50 nM of AF647-labeled monomeric FcγRIIIa at pH 7.4 and then washed with flow buffer at pH 7.4 to allow clones binding weakly at neutral pH to dissociate. The cells were then stained with PE-labeled monomeric FcγRIIIa at pH 6.5, washed with flow buffer at pH 6.5, and sorted by FACS to collect clones strongly binding at low pH (high PE and low AF647 fluorescence). Comparison of populations from each round showed enrichment for improved FcγRIIIa binding as well as pH-dependence (Fig. 3*B*).

After each round of FACS, genomic DNA was extracted from the sorted cells. The pooled Fc sequences were PCR amplified, recloned *en masse* into the Fc display plasmid, transformed into *E. coli* and plasmids from single colonies sequenced. Analysis of 23 colonies isolated from the third sorting round (R3) and 16 colonies from the fourth sorting round (R4) revealed several unique sequences. Four variants (Fig.3, *A*, *E*, and *F* and 4*A* and Table 1) were selected for further investigation based on the frequency of their appearance in R3 and R4, with 3A dominating rounds R3 (47.8%) and R4 (50%). All four variants contained six residue changes, with convergent E233D, L234V, H268D, and L235V or D substitutions. The wild-type glycine residues were strictly conserved at positions 236 and 237, and variable residues were observed at positions 267, 296, and 298. In a prior structural study, G236 and G237 were shown to have strict psi/phi angles that cannot be achieved by other amino acids (30). These residues were previously shown to be crucial for FcγR binding (33), suggesting our selection process preserves known structural constraints.

**Figure 4:**
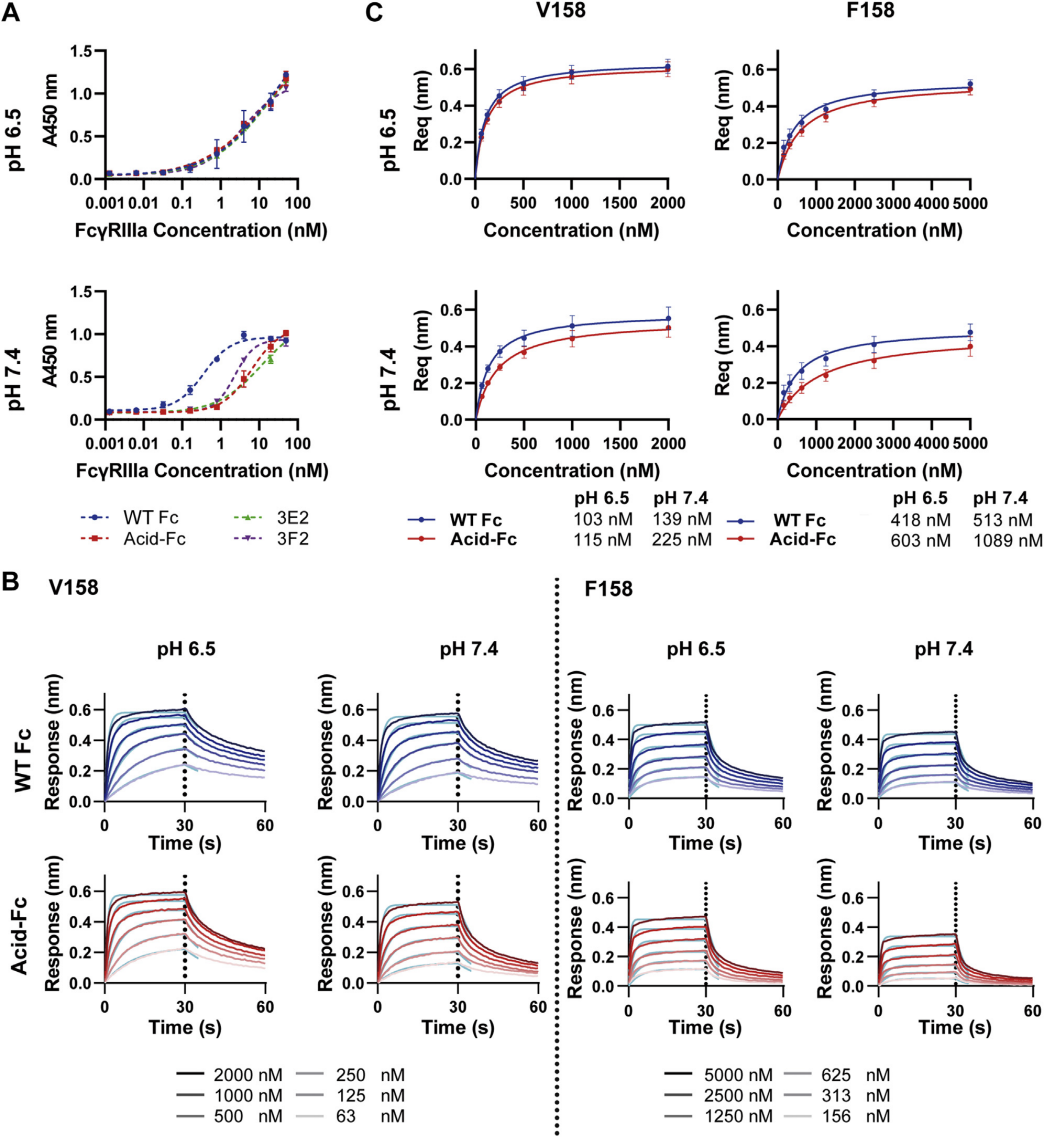
**Antibodies comprising hu4D5 Fab arms and selected Fc variants exhibit pH-selective binding to FcγRIIIa**. *A*, ELISA was performed with antibody coated at 2 μg/ml, followed by serially titrated FcγRIIIa V158 and detection with anti-His-HRP with all incubation and wash buffers maintained at the indicated pH. For the wild-type (WT) Fc and acid-Fc, BLI was performed using FAB2G tips to capture antibodies and then dipped into with serially diluted FcγRIIIa V158 (63–2000 nM) and F158 (156–5000 nM) using an OctRed96 instrument. *B*, initial kinetic responses for each phase were fit to a 1:1 binding model, while (*C*) equilibrium responses were fit to a Langmuir isotherm. The data are representative of four replicates. The obtained K_d_ values from kinetic and steady state analyses shown in Table 2. BLI, biolayer interferometry.

After transfection into CHO-T cells for analysis as monoclonal cell populations, all four variants exhibited similar display levels as wild-type Fc (Fig. S2*A*). Whereas wild-type Fc showed similar binding levels to FcγRIIIa at pH 6.5 and pH 7.4, binding for all variants at pH 7.4 was reduced compared to pH 6.5 (Fig. S2*B*). Variant pH-selectivity was quantified as the ratio of the percent of cells binding FcγRIIIa at pH 6.5 *versus* the percent binding at pH 7.4 such that a value >1 indicates greater binding at pH 6.5. Whereas the wild-type Fc exhibited a ratio of ∼1, indicating no pH-selectivity, all variants showed increased pH-dependence, with 4A having the highest pH-dependence when measured on the CHO cell surface (Fig. 3*C*).

### Characterization of selected Fc variants as soluble hu4D5 antibodies

To assess pH selectivity in the context of purified protein, we expressed the four Fc variants as full-length human IgG1 antibodies with human anti-Her2 hu4D5 (also called Trastuzumab) Fab arms, observing similar yields as hu4D5 with wild-type Fc. Binding of immobilized antibody to purified FcγRIIIa V158 was evaluated by ELISA at pH 6.5 and pH 7.4. No difference between binding at pH 6.5 *versus* pH 7.4 was observed for hu4D5 with a wild-type Fc when compared on the same plate (Fig. S2*C*), but all Fc variants showed greatly reduced FcγRIIIa binding *versus* wild-type at both pH values (Fig. S2*D*). This affinity loss was not apparent in the CHO display format, possibly due to avidity of high Fc display or increased hinge accessibility for FcγRIIIa in the absence of Fab arms.

Further inspection of the selected sequences led us to consider whether E233, L234, and L235 in the lower hinge region could have different properties as a part of a full-length antibody *versus* an isolated Fc domain, so that these residue changes should have been excluded from library design. The selected E233D and L234V substitutions are conservative changes shared among all four variants, while L235 interacts with FcγRIIIa residues on both Fc chains (30, 31). We therefore speculated that reversion of these changes might recover binding affinity without losing pH dependence. Accordingly, we generated a modified set of hu4D5 IgG variants with the native residues at positions 233 to 235 restored by site-directed mutagenesis (3A2 and 4A2 have identical sequences and were renamed “acid-Fc”, 3E2, 3F2; Table 1 and Fig. S3*A*). ELISA showed these new variants exhibit similar FcγRIIIa binding as wild-type at pH 6.5, measured by the 50% effective concentration (EC_50_) and reduced binding (larger EC_50_) at pH 7.4, as predicted (Fig. 4*A*).

To provide a more quantitative assessment of the pH-selective FcγRIIIa binding of the selected Fc variants and allow for selection of a lead candidate, we turned to biolayer interferometry (BLI), a technique that is particularly suitable for the moderate affinities of Fc–FcγR interactions. The ectodomain of FcγRIIIa V158 was purified from Expi293 cells by immobilized metal chelate affinity chromatography (Fig. S3*B*). This protein was then enzymatically biotinylated and captured by SA tips before dipping into wells containing one of the three hu4D5-Fc variants or hu4D5 with a wild-type Fc at each of six concentrations (62.5 nM to 2 μM) in pH 6.5 or pH 7.4 buffer (Fig. S4*A*) to determine steady-state apparent K_d_ values from Langmuir isotherms (Fig. S4*B*). All three Fc variants exhibited similar K_d_ values as the wild-type Fc for FcγRIIIa V158 at pH 6.5 and larger values than wild-type at pH 7.4. Among the three variants, acid-Fc had the highest apparent K_d,7.4_/K_d,6.5_ ratio of ∼2.5, indicating the greatest pH-selectivity, and was selected for further investigation.

### Acid-Fc exhibits pH-selective FcγRIIIa binding

To characterize the pH-selective binding of acid-Fc more carefully, we repeated the BLI experiment to collect kinetic binding data for hu4D5 with a wild-type or acid-Fc to both FcγRIIIa alleles at both pH values. To allow for regeneration of the biosensor tips, we used anti-CH1 FAB2G biosensors to capture each antibody and then dipped the sensors into wells containing FcγRIIIa V158 or F158, at concentrations from 62.5 to 2000 nM and 156 to 5000 nM, respectively (Fig. 4*B*). Equilibrium dissociation constants (K_d_) were calculated from on- and off-rates fitted to a 1:1 model using the entire association and the initial dissociation step as suggested by the instrument manufacturer (ForteBio) for Fc/Fc receptor-binding studies (34). Antibodies bearing a wild-type Fc exhibited K_d_ values of 134 ± 11.3 nM and 484 ± 96 nM for FcγRIIIa V158 and F158 at pH 7.4, respectively, similar to the expected affinity fold differences measured for these allotypes by surface plasmon resonance at pH 7.4 (17, 35). The measured K_d_ values for wild-type Fc at pH 6.5 appear slightly better (∼15–30%, not significant) than those at pH 7.4 for both alleles. By contrast, K_d_ values for acid-Fc were ∼2-fold worse at pH 7.4 than pH 6.5 for both FcγRIIIa alleles (*p* < 0.001) and ∼2-fold worse than the values measured for wild-type Fc at pH 7.4 for each allele (*p* < 0.001; Table 2). The apparent K_d,SS_ values were also obtained by steady state analysis and agree well with kinetic values (Fig. 4*C* and Table 2). Due to the technical limitations of BLI measurements from rebinding events during the dissociation step and evaporation during long incubations, as well as the complex binding profiles for FcγRs (34, 36), the reported K_d_ values are considered observed values to support comparisons between these two Fc variants.

**Table 2:** Fc binding kinetics to human FcγRIIIa

pH	Fc variant	FcγRIIIa V158				
		*k*_on_ ± SD (×10^5^ M^–1^s^–1^)	*k*_off_ (×10^–2^ s^–1^)	K_d_ ± SD (nM)	K_d,SS_ (nM)	Chi^2^_Kd,SS_ (nm^2^)
pH 6.5	Wild-type	4.5 ± 1.1	4.6 ± 1.2	102 ± 2.4	103	0.0303
	Acid-Fc	5.7 ± 1.3	7.4 ± 1.1	131 ± 9.9	115	0.0300
pH 7.4	Wild-type	4.9 ± 1.7	6.4 ± 1.9	134 ± 11.3	139	0.0384
	Acid-Fc	5.1 ± 1.9	11 ± 1.9	236 ± 47	225	0.0297

pH	Fc variant	FcγRIIIa F158				
		*k*_on_ ± SD (×10^5^ M^–1^s^–1^)	*k*_off_ (×10^–2^ s^–1^)	K_d_ ± SD (nM)	K_d,SS_ (nM)	Chi^2^_Kd,SS_ (nm^2^)
pH 6.5	Wild-type	3.0 ± 3.2	11.9 ± 1.1	401 ± 74	418	0.0356
	Acid-Fc	3.4 ± 6.7	17.7 ± 0.5	538 ± 107	603	0.0298
pH 7.4	Wild-type	2.8 ± 1.9	13.4 ± 2.3	484 ± 96	513	0.0445
	Acid-Fc	2.5 ± 4.2	22.6 ± 2.2	909 ± 154	1089	0.0347

The association constant(*k*_on_), dissociation constant (*k*_off_), and equilibrium dissociation constant (K_d_ = k_off_/k_on_) as well as the steady-state dissociation constant (K_d,SS_) were determined for hu4D5 antibodies with human IgG1 Fc or acid-Fc and measured by BLI. Mean values and SD (n = 4) are shown, except for K_d,SS_ values for which the Chi^2^ values from the fit are shown.

### Acid-Fc exhibits similar *in vivo* clearance rates and phagocytosis as wild-type Fc

Fc engineering can introduce destabilizing and other undesirable effects, such as altered FcRn binding and pharmacokinetics (37). Accordingly, we evaluated the biophysical characteristics of these hu4D5-Fc variants. The observed molecular weights and sizes are similar to wild-type as assessed by SDS-PAGE gel and size exclusion chromatography (Figs. S3*A* and S5*A*). Acid-Fc was somewhat destabilized, as shown by a 4.4 ^°^C lowered melting temperature as compared to wild-type (Fig. S5*B*).

Antibody *in vivo* half-life is largely determined by pH-selective binding between the Fc domain and FcRn. The acid-Fc mutations S267E, H268D, and Y296H are not in close contact (<5 Å) with FcRn or β2m residues in the cocrystal structure (38) nor have changes at these locations been previously reported to impact FcRn binding. To provide an initial assessment of acid-Fc binding to FcRn, ELISA was used to compare the binding of GST-tagged human FcRn-β2m to an antibody-coated plate at pH 6.0 or pH 7.4. As expected, acid-Fc IgG showed similar binding to FcRn as wild-type 4D5 on ELISA (Fig. S6).

**Figure 5:**
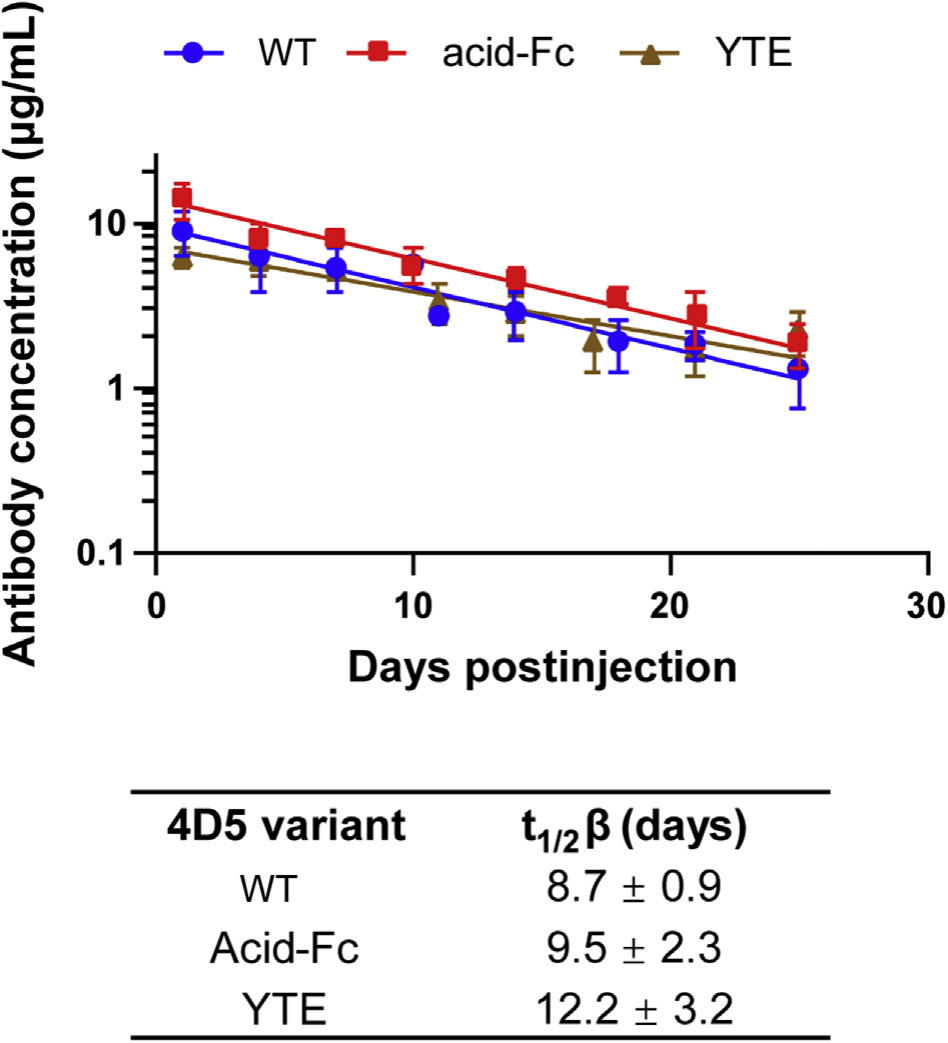
**Pharmacokinetics of Fc variant antibodies.** Homozygous Tg32 mice (n = 4 for wild-type (WT) and acid-Fc; n = 3 for YTE, female and male) expressing the human FcRn under the human promoter were administered 2 mg/kg of each antibody intra-peritoneally after which tail vein samples were collected every ∼3 days. Serum concentrations of the administered human antibody were determined by antigen-specific ELISA and plotted against time to determine the beta elimination rate constant β. Serum elimination half-lives were determined from the measured rate constants as t_1/2_β = ln2/β.

To provide a more rigorous assessment of FcRn binding behavior and the potential impacts of reduced acid-Fc thermostability *in vivo*, we used homozygous Tg32 mice which express the human FcRn under the human promoter and are often used to evaluate antibody clearance rates (39). Serum beta clearance of hu4D5 variants combined with wild-type, acid-Fc, or the M252Y/S254T/T256E (YTE) substitutions which extend *in vivo* half-life (40) were assessed. Mice were administered 2 mg/kg of each antibody intraperitoneally and subsequent serum antibody concentrations determined by antigen-specific ELISA and plotted against time to determine the beta elimination half-life (Fig. 5). As expected, the YTE variant exhibited increased t_1/2_ as compared to wild-type (∼1.4-fold). Despite having a lowered melting temperature, acid-Fc showed a t_1/2_ of 9.5 ± 2.3 days, similar to that observed for wild-type Fc (8.7 ± 0.9 days). Power analysis indicates that groups of 118 mice would be required to detect differences between these two groups with confidence at α = 0.05, suggesting the residue changes do not significantly impact *in vivo* stability.

**Figure 6:**
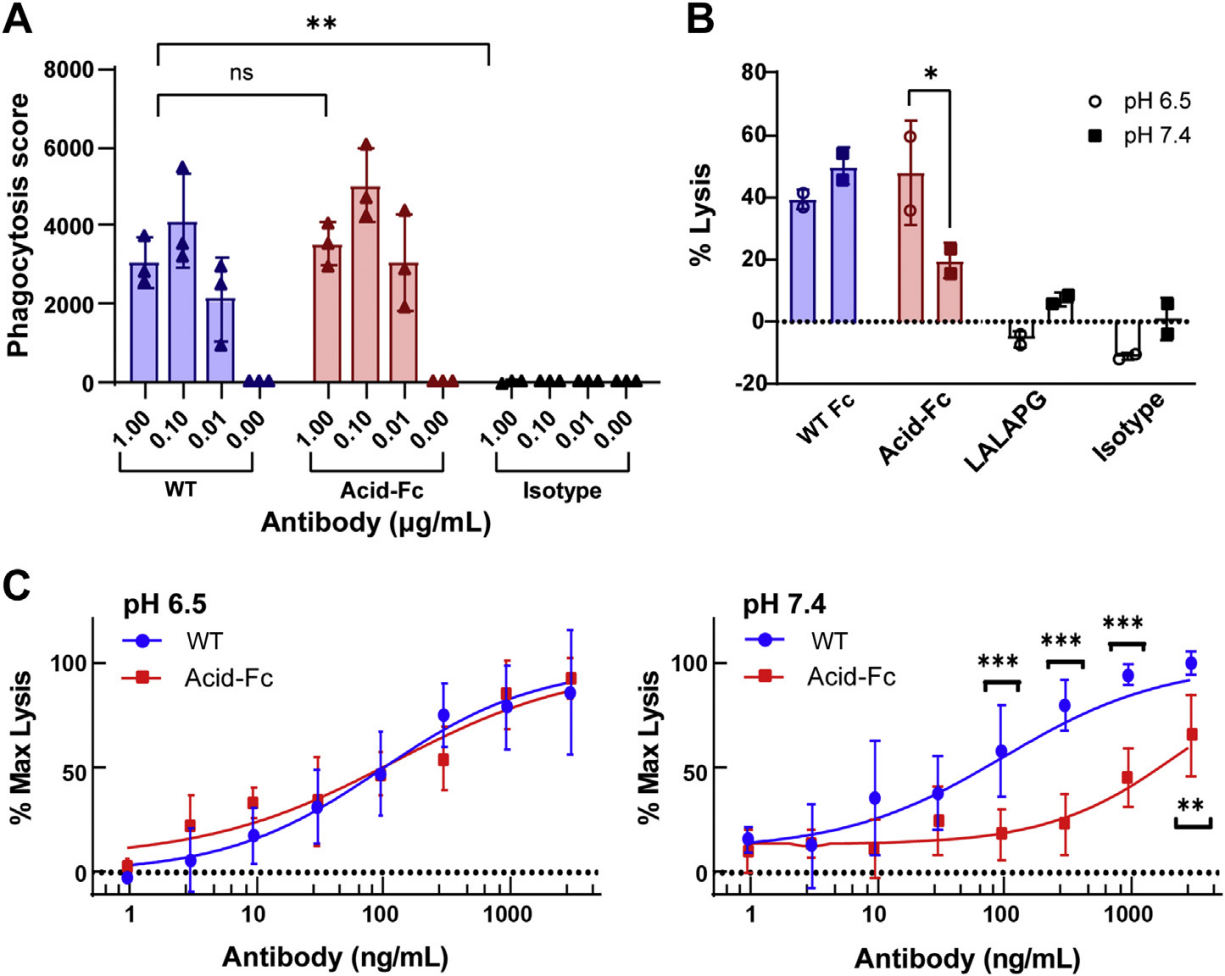
***In vitro* ADCP and ADCC mediated by Fc variant antibodies.***A*, flow cytometric ADCP assay with human THP-1 monocytic cells at pH 7.4. Her2-coated fluorescent beads were labeled with an intracellular pH fluorogenic probe before incubation with THP-1 cells and hu4D5 antibodies bearing wild-type (WT) Fc, acid-Fc, or an isotype control with wild-type Fc and an irrelevant Fab domain. After incubation, the cells were scanned by flow cytometry and the phagocytosis score calculated as %(bead internalization) ∗ GMFI(bead association). *B*, antibody variants (50 ng/ml) were added to calcein-loaded Her2-positive SKOV3 target cells in the presence of NK92 cells stably expressing FcγRIIIa (V158) for 4 h at an E:T ratio of 10:1. The data shown are representative of replicate experiments. *C*, dose-dependent ADCC assay with hu4D5 antibodies and calcein-loaded Her2-positive SKBR3 target cells in the presence of NK92 (V158) cells. The data shown are pooled from two experimental replicates, each performed with two technical replicates. Mean and SD are shown for each data point (∗*p* < 0.05; ∗∗*p* < 0.01; ∗∗∗*p* < 0.001 determined by *t* test in GraphPad), as well as the fitted four-parameter logistic curves (*smooth line*). ADCC, antibody-dependent cellular cytotoxicity; ADCP, antibody-dependent cellular phagocytosis.

To evaluate the impact of acid-Fc changes on other Fc receptors, we determined the ability of hu4D5 with wild-type or acid-Fc to mediate antibody-dependent cellular phagocytosis (ADCP) using a flow cytometric assay (41). This used the human monocytic THP-1 cell line that expresses FcγRI, FcγRIIa H131, and FcγRIIb but not FcγRIIIa (42, 43, 44) and Her2-coated fluorescent beads that were also labeled with pHrodo, a dye that only fluoresces in the low pH of the endo-lysosome, to distinguish between adherent and internalized beads. After incubation with beads and cells, both antibody variants mediated ADCP, with no significant differences in phagocytosis scores at the three antibody concentrations tested (Fig. 6*A*).

### Acid-Fc mediates pH-selective ADCC activity

To assess pH-selective activation of FcγRIIIa effector functions, we next performed a cell-based ADCC assay. ADCC is triggered by binding of FcγRIIIa on an effector cell to clustered Fc domains whose Fab arms are bound to antigens on a target cell surface. Accordingly, ADCC activity was evaluated using Calcein-loaded SKOV3 ovarian carcinoma target cells and human NK-92 effector cells stably expressing FcγRIIIa V158 (Fig. 6*B*). An antibody dose used within the dose-response range was selected (50 ng/ml) for SKOV3 cells with moderate Her2 expression (∼10^5^/cell) (25). In this experiment, hu4D5 with acid-Fc mediated lysis of ∼47.6 ± 16.8% target cells at pH 6.5, similar to that achieved by wild-type Fc at pH 7.4 (49.8 ± 5.9%) and pH 6.5 (39.1 ± 3.2%). However, at pH 7.4, acid-Fc mediated ∼2.4-fold reduced target cell lysis (19.5 ± 5.6%, *p* < 0.05), consistent with the pH-selective FcγRIIIa binding data.

To compare pH-selective ADCC activity more rigorously, we repeated the ADCC assay with SKBR3 target cells and NK-92 effector cells expressing FcγRIIIa V158 in the presence of serially diluted antibody to assess the entire dose-response curve. This allowed us to compare EC_50_ values as a more rigorous metric than differences in percent lysis at a single antibody concentration (Fig. 6*C*). For this experiment, we used SKBR3 cells, a breast carcinoma cell line with high Her2 expression (∼10^6^/cell), characteristic of aggressive tumors (25). Analysis of pooled data from replicate experiments showed minimal pH-selectivity for wild-type Fc: EC_50_ values of 96.28 ng/ml at pH 6.5 *versus* 101.4 ng/ml at pH 7.4 were measured, with overlapping 95% confidence intervals (CIs). By contrast, the acid-Fc exhibited similar efficacy at pH 6.5 as the wild-type Fc, but ∼19-fold reduced activity at pH 7.4: EC_50_ values of 120.7 ng/ml at pH 6.5 and 2307 ng/ml at pH 7.4 were measured, with nonoverlapping 95% CIs (Table 3).

**Table 3:** Antibody-dependent cellular cytotoxicity mediated by Fc variants

4D5 variants	EC_50_ (ng/ml)				
	pH 6.5	95% CI	pH 7.4	95% CI	Selectivity (pH 7.4/pH 6.5)
**WT**	96.28	37.72–200.8	101.4	30.24–165.3	1.05 ± 1.03
**Acid-Fc**	120.7	25.99–437.0	2307	813.7–6926	19.1 ± 2.5

Four-parameter logistic curves were fit to the ADCC dose-response curves with GraphPad to obtain the EC_50_ values and 95% confidence intervals shown. Selectivity was calculated by EC_50_ at pH 7.4 divided by EC_50_ at pH 6.5.

**Figure 7:**
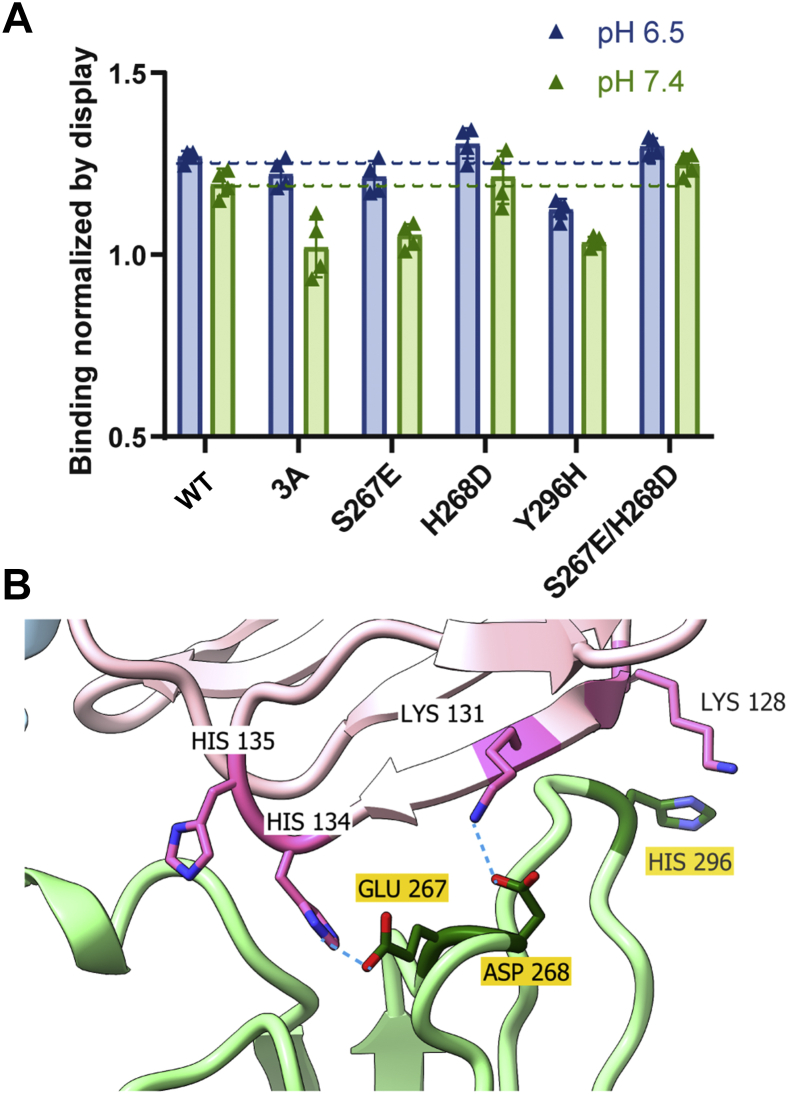
**Contributions of acid-Fc residue changes to pH selectivity.***A*, the three residues altered in acid-Fc were introduced into wild-type (WT) Fc in various combinations and displayed on the CHO cell surface alongside control Fc domains before staining to detect specific FcγRIIIa binding at pH 6.5 and 7.4 by flow cytometry. Specific FcγRIIIa binding was determined as the percent of cells positive for binding to monomeric FcγRIIIa (V158) at 50 nM divided by the percent of cells positive for Fc display. The data shown represent the mean and SDs of two experimental repeats, each performed in duplicate. *B*, possible structural mechanism by which acid-Fc changes mediate pH selectivity. The Fc/FcγRIIIa structure (PDB 3SGJ) was modified to depict the acid-Fc residue changes using the most common rotamer in ChimeraX. Under acidic conditions, Fc S267E may form electrostatic interactions with protonated H134 on FcγRIIIa, while at both pH values, binding strength may be tuned by a salt bridge formed between Fc H268D and K131 on FcγRIIIa and Fc Y296H disruption of interactions with K128 on FcγRIIIa. CHO, chinese hamster ovary.

Finally, to better understand the relative contributions of the selected residue changes to pH-selectivity, we introduced the three acid-Fc changes (S267E, H268D, and Y296H) individually and the double variant (S267E + H268D) into the wild-type Fc using site-directed mutagenesis. These variants were expressed on the CHO cell surface, alongside wild-type Fc and acid-Fc’s parent clone 3A, and assessed for FcγRIIIa binding at both pH values by flow cytometry (Fig. 7*A*). The S267E change showed reduced FcγRIIIa binding compared to wild-type only at pH 7.4, exhibiting similar pH-selectivity to that of 3A. The H268D change increased FcγRIIIa binding at both pH values while the Y296H change decreased the binding to FcγRIIIa at both pH values as compared to wild-type Fc, without affecting pH-selectivity. Surprisingly, when S267E and H268D were introduced simultaneously, overall binding was improved but pH-selectivity was lost. We speculate that the affinity gain from H268D is countered by the affinity loss due to Y296H, and together these changes tune the pH-selective interactions mediated by S267E (Fig. 7*B*).

## Discussion

In this study, we used mammalian cell display to identify human IgG1 Fc variants with pH-selective binding to FcγRIIIa and activation of ADCC. Whereas the wild-type Fc shows minimal pH-selective FcγRIIIa binding, acid-Fc contains three residue changes which reduce FcγRIIIa affinity ∼2-fold at pH 7.4 due to faster dissociation rates, but not at pH 6.5 (Fig. 4 and Table 2). These affinities were measured with purified proteins and calculated using a 1:1 stoichiometry, but the physiologically relevant interaction involves ∼10^5^ FcγRIIIa receptors expressed on an NK cell (45). FcγRIIIa signaling is triggered after binding antibody Fc domains that are clustered due to their Fab arms recognizing multiple adjacent ligand molecules on the target cell surface (46). Since this is a complex and multivalent binding interaction, cellular effects are difficult to extrapolate from measurements with purified proteins. To assess the biological activity of our engineered acid-Fc, we used *in vitro* ADCC assays with human NK-92 effector cells, anti-Her2 antibodies, and Her2-positive cells (Fig. 6, *B* and *C*). Whereas the wild-type Fc exhibited minimal pH-selective ADCC, acid-Fc demonstrated nearly 20-fold weaker activity at pH 7.4 than pH 6.5 without affecting *in vitro* ADCP activities or pharmacokinetics in Tg32 mice (Fig. 5).

To identify acid-Fc, we used a mammalian display platform that enabled us to screen Fc variants in the presence of the native glycan. This is an advantage not shared by yeast and bacterial display systems and one of the reasons why many prior Fc engineering efforts employed screening of individual point variants (47) or computational design strategies (25). The presence of the native sugar during high-throughput selection is especially relevant for Fc engineering because carbohydrate moieties occupy ∼21% (261 Å^2^) of the total Fc-FcγRIIIa interface area (31). Glycosylation at residue N297 stabilizes the Fc region in an “open” conformation, which is critical for binding to FcγRs (37). Selection of variants in the presence of different glycosylation profiles, for example, the hyper-glycosylation provided by yeast, may not be predictive of final antibody characteristics when expressed in mammalian cells, especially when sugar-proximal residues are altered. Fc variants that recapitulate this open state in the absence of glycosylation have been identified that are compatible with yeast and bacterial display (48, 49, 50, 51), but this imposes additional constraints on the variants selected. By contrast, Fc selection and production in CHO cells is expected to maintain glycoform profiles that are more consistent with profiles on mAbs expressed in CHO cells.

Mammalian display systems for antibody Fc engineering have been previously reported. A lentiviral-based mammalian display platform was recently reported by Chen *et al* (52), who screened >10^4^ Fc variants in HEK293T cells to identify Fc variants with enhanced FcγRIIIa (∼10-fold improved K_d_) and FcγRIIb binding (∼2.6-fold improved K_d_) as well as enhanced cellular activities. In our study, we were able to screen 7 × 10^6^ individual Fc-displaying CHO cells expressing ∼6 × 10^6^ unique sequences using an episomal system. CHO cell display has the additional advantage that selected proteins are expected to be compatible with current large-scale manufacturing processes since CHO cells are used to manufacture most Fc-based therapeutics (53). Future efforts to improve this platform will reduce the Fc display level to mitigate avidity impacts during selection, as well as to mimic the hinge flexibility and accessibility of an intact immunoglobulin by displaying a single-chain variable fragment-Fc fusion.

Our lead acid-Fc variant includes three Fc residue changes that contribute to pH-selective behavior: S267E, H268D, and Y296H (Table 1). An S267A substitution was previously shown to have no impact on FcγRIIIa binding (54), whereas the S267E change reported here could form electrostatic interactions with protonated H134 and H135 residues on FcγRIIIa (Fig. 7*B*). An H268D change was previously reported to directly support electrostatic FcγRIIIa interactions and indirectly influence side chain conformations of adjacent Fc residues (47). When this native Fc histidine is protonated at low pH, H268 may reduce FcγRIIIa binding by charge–charge repulsion, but replacement with a negatively charged aspartic acid may support electrostatic interactions with FcγRIIIa K131 at pH 6.5 and 7.4 (Fig. 7*B*). Finally, a new histidine was introduced at position Y296. This does not appear to mediate pH-selective interactions but may instead disrupt interactions normally formed between Y296 and FcγRIIIa K128 at both pH values so that binding is more sensitive to other pH-dependent interactions mediated by S267E. Analysis of single and double residue variants is consistent with these interpretations (Fig. 7*A*). Overall, this analysis provides a structural rationale to explain the pH-selective Fc-FcγRIIIa binding observed.

Effector cell activation mediated by FcγRIIIa requires the high-avidity crosslinking of antibody-coated target cells with effector cells because of the weak Fc-FcγRIIIa affinity (∼200–400 nM for the V158 allele or 850–4500 nM for the F158 allele) (17, 18). As a result, cellular assays are more physiologically relevant than affinities measured with soluble proteins, and modest changes in FcγRIIIa binding affinity can result in larger increases in efficacy. For example, a previously reported Fc variant with ∼10-fold tighter K_d_ led to ∼100-fold more sensitive ADCC (55). Accordingly, we performed *in vitro* ADCC assays using human NK92 cells stably expressing the high-affinity V158 FcγRIIIa allele in the presence of cell lines with high or medium Her2 expression levels to mimic clinical variation (∼10^6^/cell for SKBR3 and ∼10^5^/cell for SKOV3 cells (25)). This resulted in ∼2.4-fold increased percent of lysed cells when using one antibody concentration and SKOV3 cells (Fig. 6*B*) and nearly 20-fold pH-selectivity based on analysis of the full antibody dose-response curves with SKBR3 cells (Fig. 6*C*). A possible explanation for the observed nonlinear relationship between Fc/FcγRIIIa affinity and effector functions is that the acid-Fc residue changes alter the dynamic interactions between NK cells and target cells, which can impact ADCC through different levels of serial killing and kinetic efficiency (56).

Strategies to target drug activity to the tumor microenvironment have been previously reported, but expanding these approaches to Fc engineering is a new concept. Prior efforts include site-specific drug delivery approaches that respond to acidic pH, including peptides, liposomes, micelles, polymeric nanoparticles, and polymersomes (57), as well as antibodies with selective activity in the presence of tumor-specific proteases or small molecules such as extracellular adenosine triphosphate (58, 59). The premise that pH-selective proteins can selectively target tumors *in vivo* was first reported for the endogenous immune checkpoint molecule V-domain immunoglobulin suppressor of T cell activation (VISTA), which is rich in histidine residues and suppresses immune responses by binding the P-selectin glycoprotein ligand-1 to trigger immune-inhibitory signals only at low pH. Monoclonal antibodies preferentially binding VISTA at low pH, but not non-pH–selective antibodies, accumulated in the tumors of mice expressing human VISTA (11). Engineering to increase antibody affinity for a tumor-associated antigen at low but not neutral pH was reported in greater detail by Sulea *et al* (15). This work used structure-based computational histidine mutagenesis to guide engineering of the low affinity Her2-binding antibody bH1. Antibody variants increased pH-selectivity from 0.23 for bH1 to 5.8 as measured by pH 7.4/pH 5.0 K_d_ ratios, with the drawback that the most selective variants attained only a modest 50 nM Her2 affinity at pH 5.0, *versus* 13 nM for the parent bH1 antibody. In this work, we achieved similar K_d_ selectivity and ADCC ratios in an antigen-agnostic manner by modifying the Fc domain.

Antibodies with increased tumor-selectivity have the potential to mitigate the “on-target, off-tumor” side effects common to many antibody therapeutics. A shared characteristic which distinguishes many tumor types from healthy tissues is acidity (7, 8, 13, 14), suggesting antibodies with pH-selective activity could provide a secondary means of selective antibody activation. Future *in vitro* experiments with target cell lines ranging in Her2 expression levels and primary human effector cells will help to clarify conditions resulting in the greatest differential ADCC and explore the impact of selected Fc changes on other Fc functions. Although mapping of tumor acidosis *in vivo* in humans and mice supports the presence of appropriate pH values for even small metastases (14, 60), animal modeling will be essential to determine the feasibility of our approach. However, evaluating *in vivo* selectivity for acid-Fc is complicated by species mismatch between a human Fc and mouse FcγRs (61) and the need for ligand expression at endogenous and tumor sites; accordingly, this will be the focus of future efforts. Overall, these data support the feasibility of Fc domains with pH-selective activity as a strategy to restrict ADCC to tumor tissues and the continued investigation of this approach.

## Experimental procedures

### Cloning for Fc display on CHO-T cells

The hinge, CH2, and CH3 regions of the human IgG1 heavy chain were PCR amplified by Q5 Polymerase (New England Biolabs #M0491S) from the AbVec-hu4D5 plasmid (20) using primers #1 and #3. The PDGFR sequence (62) was amplified from pPyFab display (20) with primers #4 and #5. The two PCR products were annealed, amplified with primers #5 and #2, then introduced into the pPyEBV plasmid (63) using the *Kpn*I and *BamH*I restriction sites to create the pPy-FcDisp plasmid. Primers are listed in Table S1.

### Flow cytometry scanning of CHO-T cells displaying Fc

CHO-T cells were grown in CHO-S-SFM-II media supplemented with 2 × Glutamax (Gibco #35050061). 4.5 × 10^6^ cells were transfected either with 12.5 μg of blank pPyEBV or pPy-FcDisp plasmids using Lipofectamine 2000 (Thermo Fisher Scientific #11668500) following manufacturer’s instruction. Cells were spun down and resuspended into new media 1 day after transfection. Two days after transfection, cells were washed with 1 ml flow buffer (OptiMEM +0.5% bovine serum albumin (BSA)) and incubated for 30 min at 4 °C with either 1:1000 goat–anti-human Fc-Alexa Fluor 647 or 50 nM FcγRIIIa-SA PE.

The monomeric FcγRIIIa reagent for cell staining was generated by incubating biotinylated FcγRIIIa (V158; Sino Biological #10389-H27H1-B) with fluorescent SA overnight at 4 °C. To generate a monomeric reagent, a molar ratio of 1:7:2 FcγRIIIa: biotin: SA was used, so that <10% of the final product is expected to have >1 FcgRIIIa per SA, based on a Poisson distribution. Samples were washed, resuspended in flow buffer, and scanned by flow cytometry using a BD Fortessa. The data were analyzed in Flowjo v10.7.1, live cell gates were drawn based on FSC *versus* SSC profiles, and only this population was used for determination of mean fluorescence intensity values.

### Creation of the Fc library

Diversity was introduced into the Fc at 10 positions as indicated in Table 1 using degenerate overlapping 40-mer DNA oligomers (Sigma-Aldrich; primers in Table S1) spanning from the *KpnI* restriction site before the signal sequence to an *Xho*I restriction site within the Fc gene. The *Xho*I restriction site (CTCGAG) which was introduced into the genes coding for Fc residues P343/R345/E346 (CCTCGAGAA). Assembly PCR with 4 μM of each 40-mer and Q5 Polymerase (New England Biolabs) was used to create the mutated Fc fragments, and the assembled DNA was amplified with primers FCLibF01 and FCLibR12 by Q5 Polymerase, gel purified and digested, then ligated into pPy-FcDisp using the *Kpn*I and *Xho*I restriction sites.

### Screening of the Fc library

Library plasmid DNA, blank pPyEBV, pPyFcDisp, pPyFcDisp-LALAPG were transfected to CHO-T cells doped with a blank carrier plasmid as previously described (20). Cells were then grown at 37 °C overnight, spun at 200*g* for 5 min, and resuspended in fresh media (CHO-S-SFM-II media supplemented with 2 × Glutamax). Two days after transfection, cells were collected again and resuspended in fresh media containing 150 μg/ml hygromycin B to maintain the episome. Five days after transfection, fresh media containing 300 μg/ml hygromycin B was provided and maintained for subsequent steps. The cells were cultured for 2 weeks of growth to allow killing of cells lacking pPy episomes, while maintaining total cell numbers >5 times the library size. Two weeks after transfection, cells were collected and subjected to FACS. All staining and sorting were performed at 4 °C. For the first round of FACS, 7 × 10^6^ cells were screened for binding to 50 nM FcγRIIIa-SA-PE. After sorted cells had grown up, 1 × 10^7^ cells were screened for binding to 20 nM FcγRIIIa-SA-PE. For the last two rounds, 5 × 10^6^ cells were subjected to a dual staining process prior to FACS (Fig. S1). Cells were first stained with 50 nM monomeric FcγRIIIa-SA-AF647 in flow buffer (OptiMEM +0.5% BSA) at pH 7.4 for 30 min. The cells were then spun down and washed by flow buffer at pH 7.4. After washing, the cells were incubated with 20 nM monomeric FcγRIIIa-SA-PE in flow buffer (OptiMEM +0.5% BSA) adjusted to pH 6.5 for 30 min, washed with flow buffer at pH 6.5, and subjected to FACS. In the first two rounds of FACS, all cells with PE signal higher than that observed for the negative control LALAPG Fc were sorted into warm media and grown for 7 days. For the following two rounds of sorting, cells with PE signal greater than that observed for wild-type at the same AF647 signal were collected.

### Isolation, expression, and purification of Fc variants with hu4D5 Fab arms

After each round of cell sorting and growth, ∼10^6^ cells were collected for DNA purification using a Genomic DNA Purification Kit (Invitrogen #K182002). This was then used as a template to amplify the randomized Fc region using primers FCLibF01 and FCLibR12 and ligated into the pPyFcDisp backbone *via Kpn*I and *Xho*I restriction sites. After transformation into *E. coli*, 39 individual colonies were isolated for sequencing. To express Fc variants as full-length antibodies, the entire hinge, CH2, and CH3 region were amplified from pPyFcDisp and inserted into AbVec vector (20) encoding the hu4D5 heavy chain using Gibson assembly. After sequence confirmation, the plasmid was midi-prepped (Zymo Research#D4200) and cotransfected with plasmid encoding the hu4D5 light chain at a 1:1 ratio into 25 ml of ExpiCHO cells following manufacturer’s instruction. After 7 days expression, media were harvested and antibody was purified by protein A followed by preparative size-exclusion chromatography on a Superdex S200 column on an Åkta FPLC.

### Characterization of hu4D5-Fc variant binding to human Fcγ receptors

To express human Fcγ receptors, pcDNA3.1 plasmids containing the genes with N-term AVI and C-term His tags were transfected into ExpiHEK cells using manufacturer instructions and purified by immobilized metal affinity chromatography (Qiagen #30210). The FcγRIIIa V158 and F158 receptors were biotinylated using BirA (Avidity) and further purified by Superdex S200 size-exclusion chromatography column with an Åkta FPLC. Plasmids and GST-tagged FcRn proteins (64) were provided by George Georgiou, University of Texas at Austin.

For ELISA, 96-well high-binding plates were coated with 2 μg/ml antibody in PBS at 4 °C overnight. Wells were then blocked using 5% BSA in PBS with 0.05% Tween-20 (PBS-T) at room temperature for an hour, washed, then incubated with duplicate serial dilutions of FcγRIIIa in PBS-T adjusted to pH 6.5 or 7.4 for an hour. Wells were washed three times using PBS-T at the specified pH and captured FcγRIIIa detected with 1:1000 anti-His-HRP (Genscript Biotech #A00612). After another one-hour incubation and triplicate PBS-T wash, 50 μl TMB substrate (Thermo Scientific) was added per well followed by 50 μl of 1N HCl to quench the reaction and the absorbance at 450 nm recorded on a SpectraMax M5. For FcRn ELISA, anti-FLAG-HRP (Sigma-Aldrich #A-8592) was used for detection. The data were fit to four-parameter curves with Graphpad.

For affinity measurements *via* BLI using SA biosensors, tips were prewetted in PBS for 10 min, then dipped into wells containing 1 μg/ml monomeric biotinylated FcγRIIIa in PBS until a shift of >0.25 nm was achieved. The sensors were then dipped into wells containing kinetic buffer (PBS +0.02% Tween20 + 0.1% BSA) adjusted to pH 7.4 or 6.5 for 180 s. Antibody association signals were recorded by dipping sensors into wells containing kinetic buffer and hu4D5-Fc variants in concentrations ranging from 62.5 nM to 2 μM for 60 s. Dissociation signals were recorded by dipping sensors into wells with kinetic buffer for 120 s. For affinity measurements *via* BLI using FAB2G biosensors, antibody variants were captured on FAB2G tips until shift of 3 nm was reached, and association (30 s) and dissociation (30 s) rates were measured with serially diluted FcγRs. Association and dissociation constants were fitted from 1:1 association then dissociation model in GraphPad using the full association step and the initial 5 s of dissociation. Equilibrium K_d_ values were calculated from a Langmuir isotherm: R_eq_ = R_max_∗C/(K_d_ + C) where R_eq_ is the equilibrium response at each antibody concentration C, and R_max_ is the maximum specific binding response obtained from fitting. Statistical significance was determined by *t* test in GraphPad.

### ADCP assay

Flash red fluorescent polystyrene beads (Bangs Laboratories, Inc #FSFR004) were washed three times in sterile PBS and incubated with 25 μg/ml of recombinant Her-2 (R&D systems #10126-ER-050) for 1 h at room temperature in the dark. Beads were then washed and incubated for 1 h at room temperature in the dark with PBS with 5% fetal bovine serum (FBS) and 1:100 pHrodo iFL Green STP ester (Thermo Fischer Scientific # P36013). Beads were washed again and resuspended in PBS+5% FBS, at a stock concentration of 5 × 10^8^ beads per ml. THP-1 cells (ATCC #TIB-202) were grown in RPMI-1640 media and resuspended in 96-well plate with serially diluted antibodies and described beads at bead to cell ratio of 20:1. The cultures were incubated for 4 h at 37 ^°^C and 5% CO_2_. Cells were then washed twice with flow buffer (1% FBS in PBS), resuspended, and analyzed by BD Fortessa. The bead internalization is determined by the cell fraction of double positive for FITC and APC. The phagocytosis score is calculated as: GMFI (APC) ∗ % (bead internalization).

### ADCC assay

Target SKBR3 (ATCC #HTB-30) and SKOV3 (ATCC #HTB-77) cells were cultured in DMEM medium supplemented with 10% FBS. Effector NK-92 cells stably expressing FcγRIIIa allele V158 (ATCC #PTA 6967) cells were cultured in Alpha Minimum Essential medium without ribonucleosides and deoxyribonucleosides but with 2 mM L-glutamine and 1.5 g/L sodium bicarbonate, supplemented with 0.2 mM inositol, 0.1 mM 2-mercaptoethanol, 0.02 mM folic acid, 200 U/ml recombinant IL-2, 12.5% horse serum, and 12.5% FBS. For the ADCC assay, target cells were collected by centrifugation at 300*g* for 5 min, washed in PBS, and labeled with 2 μM Calcein-AM (BD Pharmingen #564061) in DMEM at 37 °C for 30 min. Calcein-loaded target cells were washed twice and resuspended in culture media (DMEM with 10% FBS, pH adjusted to pH 6.5 or 7.4 by addition of hydrochloric acid and 20 mM of the nonvolatile buffer Mops, as suggested by Eagle) (65) and seeded at 10,000 cells/well in 100 μL in a 96-well plate. Antibody hu4D5-Fc variants were serially diluted in 20 mM Mops-buffered saline at pH 6.5 or pH 7.4 and 50 μl added per well. NK92 effector cells resuspended in the same culture media were added to the wells at 100,000 cells/well in 50 μl for a final E:T ratio of 10:1 and incubated at 37 °C and 5% CO_2_ for 4 h. Plates were then centrifuged again to remove cells from the media. Evaluation of the final pH visually and with pH paper indicated there was no pH change during the experiment. Calcein released in the media is detected by fluorescence at excitation and emission wavelengths of 485 and 525 nm, respectively. The percent of target cells lysed was calculated as follows: 100% × (E-S)/(M-S), where E is the fluorescence of experimental well, S is the fluorescence in the absence of antibody resulting from nonspecific lysis, and M is the maximum fluorescence after treatment of target cells with lysis buffer (Triton X-100 at 2% v/v, SDS 1% w/v, 100 mM NaCl, and 1 mM EDTA). For each experiment, data were normalized to the mean percent lysis for the highest antibody concentration. Curves were then fit to four parameter logistic curves in GraphPad to determine EC_50_ values and 90% CIss. Selectivity was calculated as the ratio of the EC_50_ at pH 7.4 over the EC_50_ at pH 6.5, with statistical significance determined by two-sided *t* test in GraphPad.

### Murine pharmacokinetic studies

All animal procedures were performed in a facility accredited by the Association for Assessment and Accreditation of Laboratory Animal Care International in accordance with protocols approved by UT Austin (#2019–00226) Animal Care and Use Committees and the principles outlined in the *Guide for the Care and Use of Laboratory Animals*.

Pharmacokinetic studies were performed in homozygous transgenic Tg32 mice expressing human FcRn under the human promoter (The Jackson Laboratory Cat #014565). Mice were administered 2 mg/kg of 4D5 antibody Fc variants at 5 to 6 weeks of age by intraperitoneal injection. Blood from the lateral tail vein was collected every 3 to 4 days and used in ELISA to determine the serum antibody concentration. High-binding 96-well plates were coated overnight with 0.5 μg/ml chimeric Her2-Fc (R&D Systems), then blocked with 5% milk in PBS-T and incubated with diluted serum samples (1:1000–1:100 depending on the time point) or purified hu4D5 antibody diluted with 1:100 mouse serum in duplicate. Human antibodies were detected with goat anti-human kappa light chain antibody-HRP (Southern Biotech, 1:2000 dilution). Absorbance at 450 nm was measured after application of TMB substrate and neutralization with 1 M HCl. A four-parameter fit for each standard curve was generated in GraphPad and used to quantify the anti-Her2 human antibody present. The beta-phase elimination constant (k_e_) was determined by log-linear regression of the concentration data, including at least six time points with measurable concentrations. Beta-phase half-life was determined from t_b1/2_ = ln2/k_e_. Power analysis of the observed half-lives was performed with G∗Power using alpha level of 0.05 and desired power of 0.9.

## Data availability

Raw data will be made available upon reasonable request.

## Supporting information

This article contains supporting information Figs. S1–S6 and Table S1.

## Conflict of interest

Y. L., A. W. N., and J. A. M. are inventors on a provisional U.S. patent application no. 63/288,241 (“pH-selective antibody Fc domains”). The authors declare that they have no other conflicts of interest with the contents of this article.
