# Supplementary material for: An antibody Fc engineered for conditional antibody-dependent cellular cytotoxicity at the low tumor microenvironment pH
Source: J Biol Chem. 2022 Mar 3;298(4):101798. doi: 10.1016/j.jbc.2022.101798 (PMC9006656; doi:10.1016/j.jbc.2022.101798)
Supplement: Supporting information Figures S1–S6 and Table S1 [file mmc1.docx]

**Supporting Information for:**

**An antibody Fc engineered for conditional antibody-dependent cellular cytotoxicity at the low tumor microenvironment pH by mammalian display**

Yutong Liu^1^, Alison G. Lee^2^, Annalee W. Nguyen^1*^, Jennifer A. Maynard^1*^

Departments of ^1^Chemical Engineering and ^2^Molecular Biosciences, University of Texas, Austin, Texas, USA

*Address correspondence to Annalee Nguyen, [annalee@utexas.edu](mailto:annalee@utexas.edu) or Jennifer Maynard, [maynard@che.utexas.edu](mailto:maynard@che.utexas.edu).


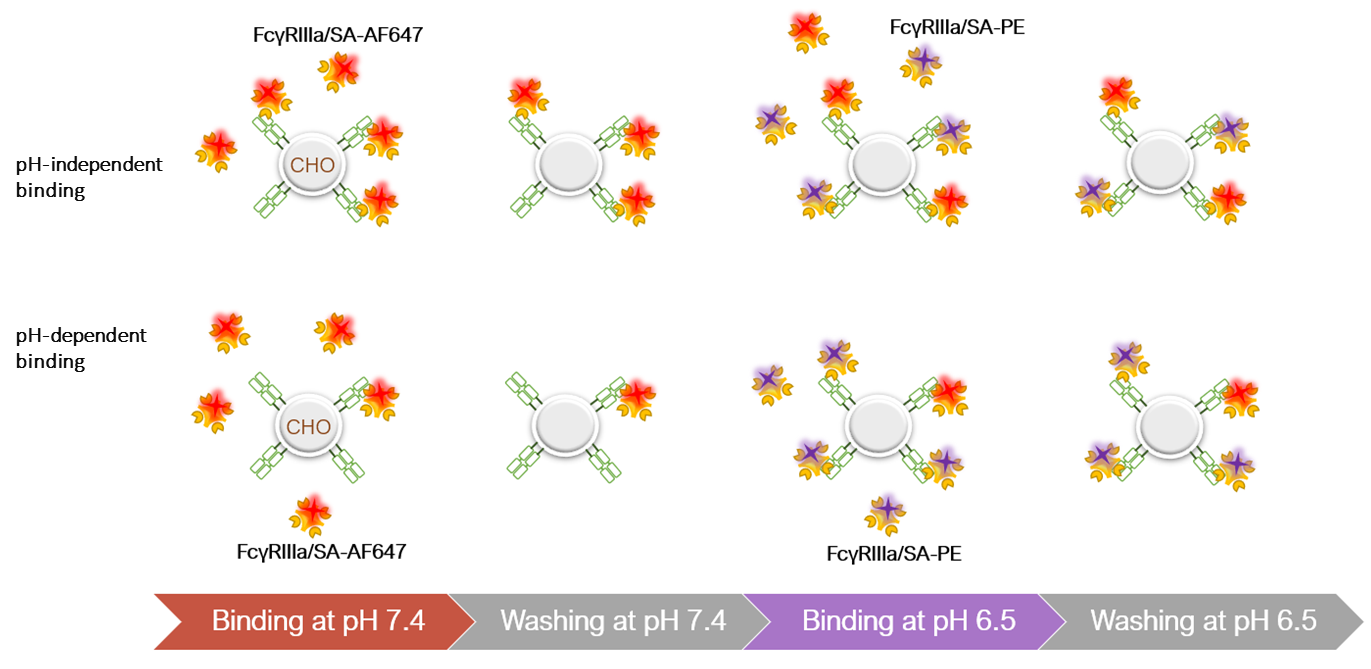


**Figure S1. Schematic of FACS selection staining strategy**. Cells were labeled with 50nM of AF647-labeled monomeric FcγRIIIa (V158) at pH 7.4, and then washed with flow buffer at pH 7.4 to allow clones binding weakly at neutral pH to dissociate. The cells were then stained with PE-labeled monomeric FcγRIIIa (V158) at pH 6.5, washed with flow buffer at pH 6.5 and sorted by FACS to collect clones with high PE and low AF647 fluorescence that preferentially bind at low pH.


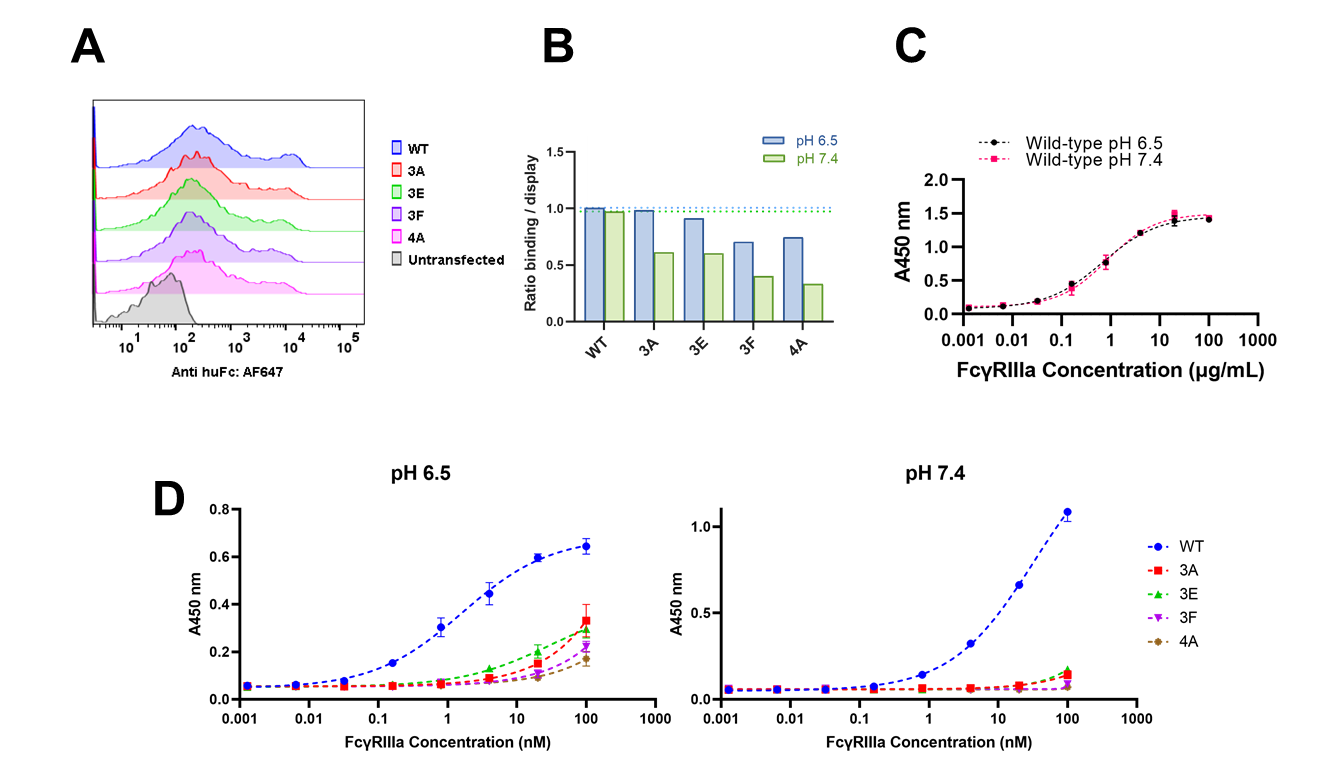


**Figure S2. Characterization of initially selected Fc variants.** (A) When single Fc variants were individually transfected into CHO-T cells for analysis on the CHO cell surface, similar Fc expression levels were observed for all variants when detected with anti-human-Fc AF647 and flow cytometry. (B) Whereas wild-type Fc showed similar binding levels to FcγRIIIa (V158)-PE at pH 6.5 and pH 7.4, binding to FcγRIIIa(V158)-PE for all variants was reduced at pH 7.4 compared to binding at pH 6.5. The percent of cells showing positive binding was normalized by the percent of cells positive for Fc display. (C, D) After reformatting as soluble IgG antibodies with hu4D5 Fab arms and Fc variants, pH-selective binding was evaluated by ELISA with immobilized antibody (2µg/mL) followed by serially titrated monomeric (V158)-FcγRIIIa with a c-terminal His-tag followed by detection with anti-His-HRP.


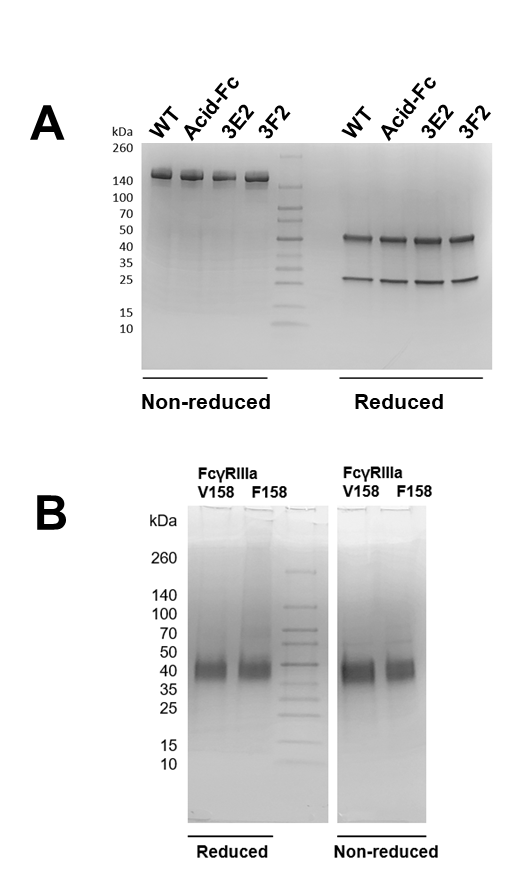


**Figure S3. Antibody and Fcγ receptor purification.** SDS-PAGE gels (4-20%) showing (A) antibodies comprised of hu4D5 Fab arms and Fc variants, and (B) recombinant FcγRs purified via c-terminal His tags. Each well is loaded with 3 μg proteins.


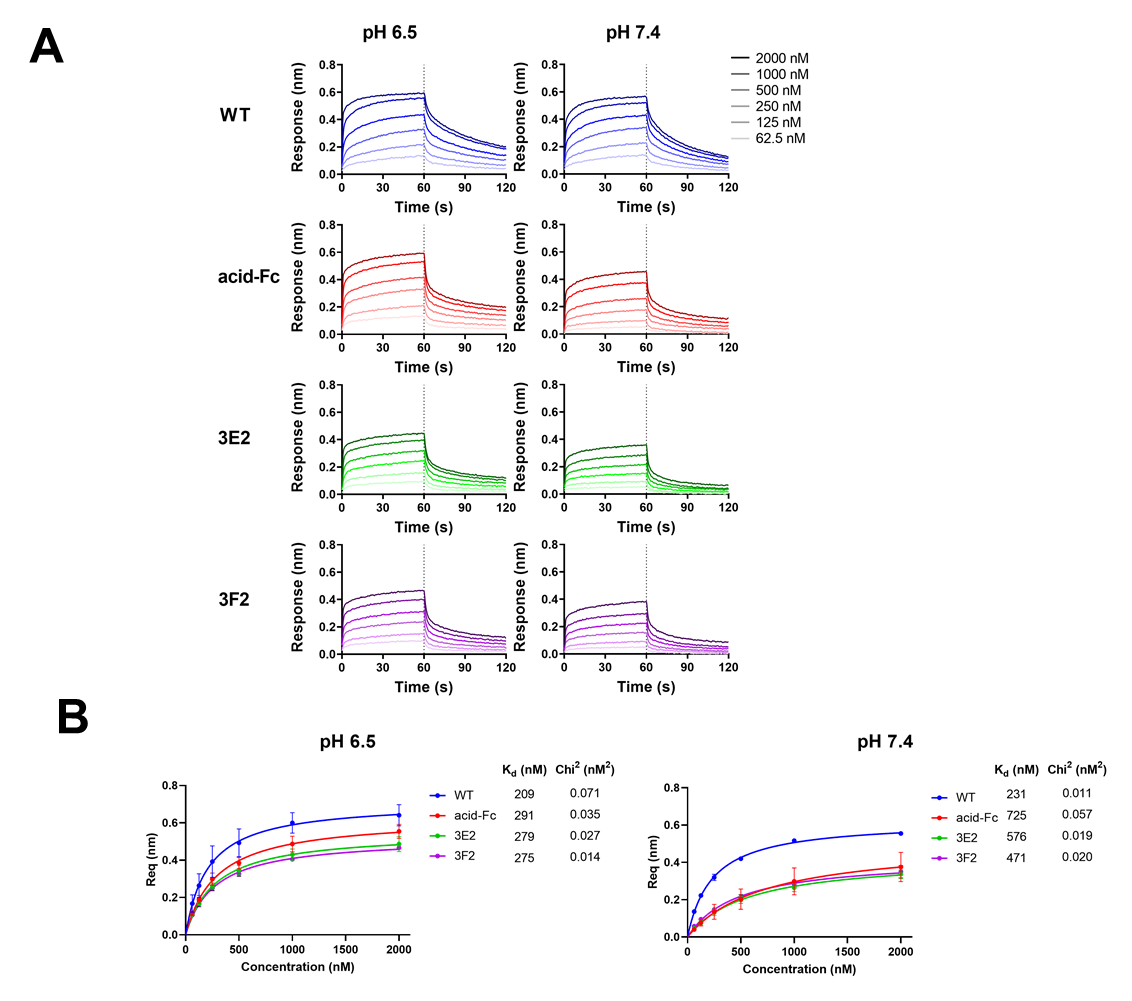


**Figure S4. Screening Fc variants for pH-selective FcγRIIIa binding by BLI.** BLI was performed by capture of biotinylated FcγRIIIa V158 on streptavidin tips which were dipped into serially diluted antibodies comprised of hu4D5 Fab arms and Fc variants (62.5 nM to 2 μM). Data shown are representative of two experimental repeats, each with six antibody concentrations. (B) The equilibrium responses were fit to Langmuir isotherms to determine equilibrium K_d_ values. Data shown represent the mean and range of two experimental repeats, as well as the fitted isotherm curves. Chi^2^ values from the fits are noted.


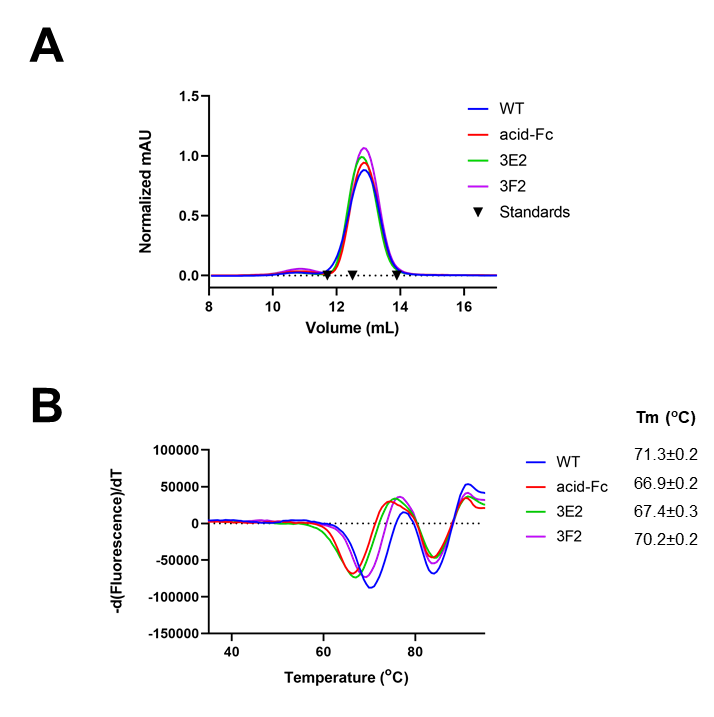


**Figure S5. Biophysical characterization of hu4D5 antibodies with variant Fcs.** (A) To assess monodispersity, antibodies were analyzed by size exclusion chromatography using a Superdex S200 column on an Åkta FPLC. Elution volumes for the molecular weight standards are indicated by triangles and include (from left to right): Beta Amylase (200kDa), Aldolase (158kDa), Conalbumin (75kDa). (B) To assess thermal stability, antibody variants (100 µg/mL) were mixed with Protein Thermal Shift™ (Thermal Fisher) dye, and the melt curve with ramp rate of 0.05 °C/sec was measured by real-time PCR using ViiA7™ machine.

**Figure S6. Binding of variant Fcs to FcRn.** ELISA was performed with antibody comprised of hu4D5 Fab arms and the indicated Fc variants at (A) pH 6.0 or (B) pH 7.4. Purified antibodies were immobilized at 2 μg/ml followed by blocking, titration of FLAG-tagged FcRn in buffer at the indicated pH and detection with anti-FLAG HRP.

**Table S1. List of primers used in this study.**

Degenerate codons used for library primers: W (A or T), S (C or G), M (A or C), K (G or T), R (A or G), Y (C or T), B (C or G or T), D (A or G or T), H (A or C or T), V (A or C or G), N (any base).

| **Primer Name** | **5’ to 3’ sequence** |
| --- | --- |
| **#1** | ATACTATTCCTGGTAGCAACCGCTACAGGAGTACATTCCGAGCCCAAATCTTGTGACAAAAC |
| **#2** | GCTCGTGGTACCGCGCGCCTAAATGGGTTGAACCATGGGCTGGTCCTGCATCATACTATTCCTGGTAGCAACCGCTAC |
| **#3** | ACCGGAACCGCCTCCTGAACCTCCACCTTTACCCGGGGACAGG |
| **#4** | GGAGGCGGTTCCGGTAATGCTGTGGGCCAGG |
| **#5** | GCTCGTGGATCCCTAACGTGGCTTCTTCTG |
| **FCLibF01** | GCTCGTGGTACCGCGCGCCTAAATGGGTTGAACCATGGGC |
| **FCLibF02** | TGGTCCTGCATCATACTATTCCTGGTAGCAACCGCTACAG |
| **FCLibF03** | GAGTACATTCCGAGCCCAAATCTTGTGACAAAACTCACAC |
| **FCLibF04** | ATGCCCACCGTGCCCAGCACCTSAWSWSSWKGRSGRWCCG |
| **FCLibF05** | TCAGTCTTCCTCTTCCCCCCAAAACCCAAGGACACCCTCA |
| **FCLibF06** | TGATCTCCCGGACCCCTGAGGTCACATGCGTGGTGGTGGA |
| **FCLibF07** | CGTGRRSBMCGAAGACCCTGAGGTCAAGTTCAACTGGTAC |
| **FCLibF08** | GTGGACGGCGTGGAGGTGCATAATGCCAAGACAAAGCCGC |
| **FCLibF09** | GGGAGGAGCAGYACAACMRCACGTACCGTGTGGTCAGCGT |
| **FCLibF10** | CCTCACCGTCCTGCACCAGGACTGGCTGAATGGCAAGGAG |
| **FCLibF11** | TACAAGTGCAAGGTCTCCAACAAAGMSCTCCCAGCCCCCA |
| **FCLibF12** | TCGAGAAAACCATCTCCAAAGCCAAAGGGCAGCCTCGAGA |
| **FCLibR01** | AATAGTATGATGCAGGACCAGCCCATGGTTCAACCCATTT |
| **FCLibR02** | TTTGGGCTCGGAATGTACTCCTGTAGCGGTTGCTACCAGG |
| **FCLibR03** | GTGCTGGGCACGGTGGGCATGTGTGAGTTTTGTCACAAGA |
| **FCLibR04** | GGGGGGAAGAGGAAGACTGACGGWYCSYCMWSSWSWTSAG |
| **FCLibR05** | CTCAGGGGTCCGGGAGATCATGAGGGTGTCCTTGGGTTTT |
| **FCLibR06** | CAGGGTCTTCGKVSYYCACGTCCACCACCACGCATGTGAC |
| **FCLibR07** | TGCACCTCCACGCCGTCCACGTACCAGTTGAACTTGACCT |
| **FCLibR08** | GYKGTTGTRCTGCTCCTCCCGCGGCTTTGTCTTGGCATTA |
| **FCLibR09** | CCTGGTGCAGGACGGTGAGGACGCTGACCACACGGTACGT |
| **FCLibR10** | TTGGAGACCTTGCACTTGTACTCCTTGCCATTCAGCCAGT |
| **FCLibR11** | TTTGGAGATGGTTTTCTCGATGGGGGCTGGGAGSKCTTTG |
| **FCLibR12** | GCAGGGTGTACACCTGTGGTTCTCGAGGCTGCCCTTTGGC |
